# Evaluation of Multiple Intravenous Infusions of Autologous Adipose‐Derived Mesenchymal Stem Cells in Parkinson’s Disease: A Randomized, Double‐Blind Clinical Trial

**DOI:** 10.1155/padi/9934417

**Published:** 2026-05-13

**Authors:** Ridhima Vij, Hosu Kim, Hyeonggeun Park, Djamchid Lotfi, Thanh Cheng, Donna Chang

**Affiliations:** ^1^ Clinical Research Division, Hope Biosciences Research Foundation, Sugar Land, Texas 77478, USA; ^2^ Cell Production Team, Hope Biosciences, Sugar Land, Texas 77478, USA

**Keywords:** adipose derived, clinical trial, efficacy, intravenous, mesenchymal stem cells, Parkinson’s disease, safety

## Abstract

**Introduction:**

The purpose of this trial was to evaluate preliminary efficacy and safety of multiple intravenous infusions of autologous, Hope Biosciences adipose‐derived mesenchymal stem cells (HB‐adMSCs) for the treatment of patients with Parkinson’s disease (PD).

**Methods:**

A total of *N* = 24 patients with PD were randomized 5:3 into HB‐adMSC (200 million) or Placebo. Six intravenous infusions of HB‐adMSCs or saline were administered at Weeks 0, 4, 8, 16, 24, and 32 with a follow‐up at Week 42 and the end of study (EOS) at Week 52. The primary efficacy endpoint was to investigate change from baseline to Week 52 in Movement Disorder Society‐Unified Parkinson’s Disease Rating Scale (MDS‐UPDRS) Part II. Primary safety endpoints included the incidence of adverse and serious adverse events (AE/SAEs) and clinically significant changes in laboratory assessments.

**Results:**

HB‐adMSC treatment was well tolerated and safe, with only one SAE reported, which was deemed unrelated to the investigational product. In total, 81 AEs were recorded, 79 of which were mild, one moderate, and one severe. Neurological disorders were the most commonly reported AEs, with a higher incidence in the placebo group (66.7%) compared with the HB‐adMSC group (46.7%). No deaths occurred, and no clinically meaningful changes were observed in laboratory parameters from baseline to the EOS for either group. Efficacy analyses revealed no clear treatment effect in MDS‐UPDRS II scores between the HB‐adMSC and Placebo groups.

**Conclusion:**

Multiple intravenous infusions of autologous HB‐adMSCs were found to be safe and feasible in the patients with PD. Efficacy outcomes should be interpreted with caution, as this small, early‐phase study was not designed or powered to demonstrate definitive efficacy.

**Trial Registration:** ClinicalTrials.gov identifier: NCT04928287

## 1. Introduction

Parkinson’s disease (PD) is the second most prevalent neurodegenerative disorder, after Alzheimer’s disease, affecting around 1‐2% of individuals aged 65 and older [1]. The condition is marked by progressively worsening motor symptoms, including bradykinesia, rigidity, tremor, and postural instability, along with nonmotor features such as cognitive impairment, sleep disturbances, and autonomic dysfunction [2]. The pathogenesis of PD is driven by the progressive loss of dopaminergic neurons in the substantia nigra, resulting in reduced dopamine levels in the striatum [3].

Currently available treatments, including dopaminergic replacement therapies and deep brain stimulation, are primarily directed toward symptomatic management and do not prevent disease progression; moreover, their long‐term use may be associated with clinically meaningful adverse effects [4–6]. In recent years, stem cell therapies have gained attention as potential therapeutic strategies for degenerative disorders due to their capacity to support tissue repair and modulate immune‐mediated injury. Mesenchymal stem cells (MSCs) have been particularly noted for their tissue repair capabilities through the release of paracrine factors, low immunogenicity, secretion of soluble factors such as interleukin 6, and suppression of T and B lymphocytes’ activation and proliferation [7, 8]. Additionally, MSCs release anti‐inflammatory and antiapoptotic molecules, potentially protecting damaged tissues [9]. In preclinical models of PD, MSCs have been reported to promote neuronal survival, modulate neuroinflammatory responses, and, in some settings, exhibit dopaminergic differentiation potential, suggesting potential therapeutic benefits in PD [10–14].

This study presents the findings of a randomized, double‐blind, placebo‐controlled trial investigating the safety and efficacy of multiple intravenous infusions of autologous, HB‐adMSCs in individuals with PD. The study aims to assess the safety and potential symptom improvement with HB‐adMSC therapy, contributing to the development and feasibility of MSC‐based disease‐modifying treatments for PD. The main objective of the study was to evaluate safety and preliminary efficacy of HB‐adMSCs over placebo in PD patients, as determined by improvements in the quality of life, measured by MDS‐UPDRSII assessment. The secondary objective was to evaluate the therapeutic efficacy to improve motor and nonmotor symptoms compared with placebo, based on changes in other MDS‐UPDRS scores, patient‐reported outcomes, and changes in the dosage of medications at the end of study (EOS).

## 2. Methods

### 2.1. Study Design

Six infusions each of either autologous HB‐adMSCs or saline were administered intravenously at the rate of 4‐5 mL/min at Weeks 0, 4, 8, 16, 24, and 32 to each of the eligible 24 subjects, randomly allocated to each of the two groups: HB‐adMSCs (200 million) or Placebo in a 5:3 ratio. The total study duration was 52 weeks (EOS). The study was approved by Western Institutional Review Board (IRB), in Olympia, Washington. All procedures were conducted in accordance with Good Clinical Practice guidelines and the Declaration of Helsinki. Written informed consent for participation was provided by all subjects.

### 2.2. Eligibility

#### 2.2.1. Inclusion Criteria

(1) Males and females aged 18–75 years; (2) must have been diagnosed with early and/or moderate PD at least 6 months before participation; (3) must have previously banked MSCs with Hope Biosciences; (4) should be able to read, understand, and provide written consent; (5) voluntarily signed informed consent before any clinical trial related procedures; (6) female subjects should not be pregnant or plan to become pregnant during study participation; (7) male subjects, if their sexual partners can become pregnant should use a method of contraception during the study; and (8) must be able and willing to comply with the requirements of the trial.

#### 2.2.2. Exclusion Criteria

(1) Pregnancy and lactation; (2) advanced PD described as severe disability, wheelchair bound, or bedridden; (3) active malignancy; (4) alcoholic addiction or dependency or substance use; (5) one or more significant concurrent medical conditions; (6) received any stem cell treatment within 6 months before the first dose; (7) received any investigational therapy or any approved therapy for investigational use within 1 year prior to the first dose other than COVID‐19 vaccines; (8) laboratory abnormality during screening, including white blood cell count < 3000/mm^3^, platelet count < 80,000 mm^3^, absolute neutrophil count < 1500/mm^3^, and ALT or AST 10 upper limit of normal x 1.5; (9) other laboratory abnormality or medical condition which, in the opinion of the investigator, poses a safety risk or will prevent the subject from completing the study; (10) unlikely to complete or adhere to the study procedures; (11) known concurrent acute or chronic viral hepatis B or C or HIV infection; (12) has a previously diagnosed psychiatric disorder; (13) systemic infection requiring treatment with antibiotics, antivirals, or antifungals within 30 days prior to the first dose; (14) male subjects who plan to donate sperm during the study or within 6 months after the last dose and female subjects who plan to donate eggs or undergo IVF treatment; and (15) determined unsuitable for study enrollment for other reasons by the investigator.

### 2.3. Autologous HB‐adMSCs Isolation and Administration

For the production of autologous HB‐adMSCs, emulsified fat was extracted from the subjects’ abdomens via liposuction performed by a licensed physician. The amount of adipose tissue varied between subjects, typically ranging from 2 to 8 mL. This tissue was then treated with collagenase to isolate the stromal vascular fraction (SVF), which was plated in Hope Biosciences’ HB‐103 medium. The resulting adherent cells were expanded in HB‐101 medium to establish a passage 0 (P0) culture. Cells were cryopreserved at passages 0, 1, and 2 to create a complete bank for each subject. Cells from passage 2 were thawed and cultured to passage 4.

A total of 2.0 × 10^8^ ± 20% HB‐adMSCs were freshly harvested from passage 4 cultures, packaged in 20‐mL sterile saline (0.9% sodium chloride), and administered within 96 h of packaging. Infusions were administered at Weeks 0, 4, 8, 16, 24, and 32.

To ensure safety and efficacy of the investigational drug, HB‐adMSCs were manufactured under current Good Manufacturing Practice (cGMP)‐compliant conditions and subjected to predefined release criteria, including viability (≥ 70%), appearance (opaque white to faint yellow with no sedimentation), USP71 sterility (no organism seen), Mycoplasma (negative), endotoxin (≤ 10EU/mL), Gram stain (no organism seen), and identity/purity by MSC‐defining surface markers CD73 and CD29 (> 75%) and CD31 and CD45 (< 5%). All products provided for the study successfully met these quality control standards. Placebos consisted of 20‐mL sterile saline (0.9% sodium chloride) prefilled in the same type of syringe as those used for HB‐adMSCs packaging and were also tested for USP71 sterility, Mycoplasma, and endotoxin to ensure safety. See Supporting Information Table S1 for quality control metrics.

The methodology used in this study for the production and administration of HB‐adMSCs follows the approach outlined by de Dios et al. [15], with the portions of the methods section partly reproducing their original wording.

### 2.4. Randomization and Blinding

Post screening, 24 eligible subjects were randomized 5:3 (treatment group:placebo) at baseline. Randomization was performed by a neutral personnel, based on stratification categories that included (1) PD severity: mild (MDS‐UPDRS score < 13 AND Carbidopa/Levodopa < 375 mg/day) or moderate (MDS‐UPDRS > 13 OR Carbidopa/Levodopa > 375 mg/day); (2) age: < 50 or > 50; and (3) sex: male or female. Treatment groups were identified as A and B. Group assignment was determined by the randomization spreadsheet, based upon the three factors mentioned above. All patients, investigators, and site staff were blinded to the assigned treatment. Amber bag covers were applied to saline infusion bags for product infusion. Only subject ID, DOB, and expiration date were on the bag label to ensure proper distribution. The mixer injected the product into the bag, covered it, and applied a label before handing it off to the study coordinators. Product was infused into an amber bag with 250 mL saline. Premixed bags were handed off to the clinical team for treatment administration.

### 2.5. Endpoints

The primary efficacy endpoint was to assess changes in MDS‐UPDRS Part II from baseline to Week 52 (EOS). Primary safety endpoints included incidence of adverse event (AE) and serious adverse events (SAEs) and clinically significant changes in laboratory values (including hematology, biochemistry, and coagulation parameters), vital signs, weight, and physical examination results.

Secondary endpoints included changes from baseline in MDS‐UPDRS Parts I, III, and IV, Parkinson’s Disease Fatigue Scale‐16 (PFS‐16), Parkinson’s Disease Questionnaire‐39 (PDQ‐39), Visual Analog Scale (VAS) for pain and muscle spasms, Neuro QoL assessments (included communication, ability to participate in social roles, anxiety, depression, emotional and behavioral dyscontrol, fatigue, lower extremity function: mobility, positive affect and well‐being, sleep disturbance, upper extremity function, stigma, satisfaction with social roles and activities, and cognition function), and changes in dosage of medications taken to treat PD.

### 2.6. Statistical Methods

*N* = 24 eligible subjects were randomized in 5:3 ratio into HB‐adMSC vs. Placebo groups. All randomized subjects who received at least one dose of HB‐adMSCs or Placebo were included in the safety analysis set. Subjects who received all six infusions of either HB‐adMSCs or Placebo were included in the efficacy analysis set. All laboratory parameters were summarized descriptively, while physical examination and vital signs were evaluated using counts and percentages based on subjects. The nonparametric Mann–Whitney *U* test (or Wilcoxon rank‐sum test) was applied to the primary and secondary efficacy endpoints to test the significance of treatment effects by comparing median values. The Bonferroni–Holm method was used for multiplicity adjustment of the efficacy endpoints. For measurements performed over time, median values were plotted to explore the trends. All hypotheses were tested at a 95% confidence interval (*p* = 0.05, two‐sided test), unless stated otherwise. Data were analyzed using SAS program Version 9.4 M7.

## 3. Results

### 3.1. Demographics and Baseline Characteristics

**FIGURE 1:**
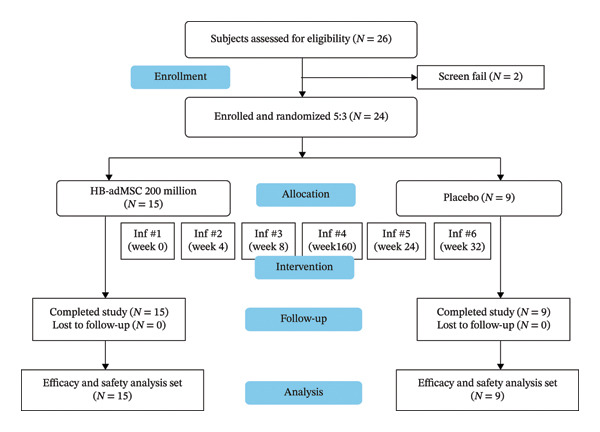
Schematic CONSORT flow diagram. A total of 24 subjects who passed screening were enrolled and randomly assigned in a 5:3 ratio to two groups: 200 million HB‐adMSC (*n* = 15) or Placebo (*n* = 9). Inf = infusion.

Out of 26 subjects assessed for screening, 24 passed and were enrolled, while 2 failed. The eligible subjects were randomly assigned to one of the two treatment groups: 200 million HB‐adMSC or Placebo to receive six infusions each at Week 0 (baseline), Week 4, Week 8, Week 16, Week 24, and Week 32, with an EOS at Week 52 (CONSORT flow diagram, Figure 1). The study population consisted of 50% females and 50% of male subjects. The majority of the population (87.5%) was non‐Hispanic or Latino, while the remaining 12.5% comprised Hispanic or Latino ethnicity. A total of 91.7% of the study participants were white. The mean age of study subjects was 60.6 (SD = 10.29) years with median 62.0 (min: 39.0 and max: 75.0) years. Mean (SD) height, weight, and BMI were 174.09 (SD = 9.02) cm with median of 175.30 (min: 154.9 and max: 190.5) cm, 79.33 (SD = 22.15) kgs with median of 82.10 (min: 45.4 and max: 123.4) kgs, and 25.83 (SD = 5.6) kg/m^2^ with a median of 26.62 (min: 17.2 and max: 38.0) kg/m^2^, respectively. The mean PD duration for the study subjects was 4.21 (SD = 3.11) with 45.8% of the subjects with mild disease severity and the remaining 54.2% were with moderate PD severity (Table 1). Detailed summary of the medical history of all subjects is provided in Supporting Information Table S2.

**TABLE 1:** Baseline and demographic characteristics by treatment group.

	**HB-adMSCs** ***N* = 15**	**Placebo** ***N* = 9**	**Overall** ***N* = 24**

Sex *n* (%)			
Female	8 (53.3)	4 (44.4)	12 (50.0)
Male	7 (46.7)	5 (55.6)	12 (50.0)
Age (years)			
Mean (SD)	60.3 (11.29)	61.1 (8.99)	60.6 (10.29)
Median (min; max)	63.0 (39; 75)	61.0 (51; 74)	62.0 (39; 75)
Race *n* (%)			
American Indian or Alaska Native	0 (0.0)	0 (0.0)	0 (0.0)
Asian	1 (6.7)	1 (11.1)	2 (8.3)
Black or African American	0 (0.0)	0 (0.0)	0 (0.0)
Native Hawaiian or other Pacific Islander	0 (0.0)	0 (0.0)	0 (0.0)
White	14 (93.3)	8 (88.9)	22 (91.7)
Ethnicity *n* (%)			
Hispanic or Latino	2 (13.3)	1 (11.1)	3 (12.5)
Not Hispanic or Latino	13 (86.7)	8 (88.9)	21 (87.5)
Height (cm)			
Mean (SD)	174.41 (8.572)	173.57 (10.239)	174.09 (9.020)
Median (min; max)	175.30 (160.0; 190.5)	175.30 (154.9; 188.0)	175.30 (154.9; 190.5)
Weight (kg)			
Mean (SD)	79.56 (23.389)	78.94 (21.280)	79.33 (22.149)
Median (min; max)	80.70 (47.6; 123.4)	83.50 (45.4; 110.7)	82.10 (45.4; 123.4)
BMI (kg/m^2^)			
Mean (SD)	25.82 (5.897)	25.85 (5.370)	25.83 (5.585)
Median (min; max)	26.52 (18.6; 38.0)	27.41 (17.2; 34.1)	26.62 (17.2; 38.0)
Parkinson’s disease duration at baseline (years)			
Mean (SD)	4.46 (3.371)	3.79 (2.740)	4.21 (3.105)
Median (min; max)	3.20 (0.6; 11.9)	3.10 (0.6; 7.5)	3.15 (0.6; 11.9)
Parkinson’s disease severity			
Mild	7 (46.7)	4 (44.4)	11 (45.8)
Moderate	8 (53.3)	5 (55.6)	13 (54.2)

Abbreviation: HB‐adMSCs, Hope Biosciences adipose‐derived MSCs.

### 3.2. Efficacy Endpoints

The primary efficacy of HB‐adMSCs vs. Placebo was assessed as changes (decreases) in MDS‐UPDRS Part II from baseline to EOS at Week 52. There were no statistically significant differences observed between the HB‐adMSC and Placebo groups (*p* = 0.575; Table 2) from baseline to Week 52.

**TABLE 2:** Efficacy endpoints.

Outcome measure median (IQR)	Baseline (infusion 1)			EOS		
	HB‐adMSCs *N* = 15	Placebo *N* = 9	*p*‐value	HB‐adMSCs *N* = 15	Placebo *N* = 9	*p* value
PDQ‐39	28.0 (9.0–42.0)	16.0 (10.0–32.0)	0.396	29.0 (7.0–42.0)	14.0 (7.0–23.0)	0.349
PFS‐16	36.0 (16.0–52.0)	38.0 (20.0–45.0)	0.906	41.0 (16.0–57.0)	21.0 (17.0–49.0)	0.496
MDS‐UPDRS I	8.0 (5.0–12.0)	7.0 (2.0–9.0)	0.535	8.0 (3.0–13.0)	8.0 (1.0–10.0)	0.835
MDS‐UPDRS II	12.0 (4.0–13.0)	9.0 (3.0–10.0)	0.362	8.0 (4.0–17.0)	5.0 (3.0–12.0)	0.575
MDS‐UPDRS III	26.0 (17.0–34.0)	21.0 (15.0–24.0)	0.348	13.00 (6.0–27.0)	20.0 (11.0–21.0)	0.929
MDS‐UPDRS IV	0.0 (0.0–6.0)	4.0 (3.0–5.0)	0.340	0.0 (0.0–4.0)	1.0 (0.0–4.0)	0.418
VAS total (pain and muscle spasm)	23.0 (10.0–54.0)	9.0 (2.0–30.0)	0.321	12.0 (0.0–50.0))	9.0 (0.0–10.0)	0.158
Neuro‐QoL						
Anxiety	17.0 (14.0–23.0)	14.0 (12.0–16.0)	0.140	14.0 (10.0–21.0)	10.0 (8.0–14.0)	0.252
Cognitive function	32.0 (29.0–40.0)	36.0 (29.0–38.0)	0.812	39.0 (30.0–39.0)	38.0 (30.0–40.0)	0.675
Communication	24.0 (21.0–25.0)	23.0 (23.0–24.0)	0.564	24.0 (21.0–25.0)	24.0 (21.0–25.0)	0.829
Depression	11.0 (8.0–15.0)	9.0 (8.0–11.0)	0.271	10.0 (8.0–15.0)	9.0 (8.0–11.0)	0.736
Fatigue	15.0 (10.0–22.0)	15.0 (10.0–16.0)	0.679	17.0 (8.0–24.0)	12.0 (9.0–20.0)	0.459
Mobility	38.0 (34.0–40.0)	40.0 (37.0–40.0)	0.186	38.0 (31.0–40.0)	40.0 (35.0–40.0)	0.317
Emotional dyscontrol	12.0 (9.0–16.0)	10.0 (8.0–12.0)	0.264	11.0 (8.0–14.0)	9.0 (8.0–9.0)	0.171
Positive well‐being	36.0 (33.0–44.0)	37.0 (36.0–44.0)	0.208	39.0 (36.0–45.0)	42.0 (28.0–44.0)	0.698
Social roles/activities	37.0 (35.0–40.0)	39.0 (31.0–39.0)	0.609	37.0 (30.0–40.0)	40.0 (32.0–40.0)	0.582
Sleep disturbance	14.0 (12.0–22.0)	14.0 (11.0–16.0)	0.498	14.0 (8.0–17.0)	11.0 (10.0–12.0)	0.392
Stigma	11.0 (8.0–16.0)	12.0 (8.0–15.0)	0.952	12.0 (8.0–13.0)	9.0 (9.0–10.0)	0.763

*Note:* *p* value calculated using the nonparametric Wilcoxon rank‐sum test.

Abbreviations: EOS, end of study; HB‐adMSCs, Hope Biosciences adipose‐derived mesenchymal stem cells (200 million); IQR, interquartile range; MDS‐UPDRS, Movement Disorder Society‐Unified Parkinson’s Disease Rating Scale; PDQ‐39, Parkinson’s Disease Questionnaire‐39; PFS‐16, Parkinson’s Fatigue Scale‐16; VAS, Visual Analog Scale.

Secondary efficacy endpoints included changes in both motor and nonmotor aspects, as well as improvements in the quality of life for PD patients. These endpoints encompassed changes in MDS‐UPDRS Parts I, III, and IV, changes in Neuro‐QoL, PFS‐16, PDQ‐39, and changes in total VAS score (pain and muscle spasms). Across all time points evaluated, there were no significant differences detected between the treatment groups for any of these secondary endpoints (*p* > 0.05; Table 2). Additionally, analysis of changes in PD medication dosages revealed no significant differences between the two groups.

### 3.3. Safety Endpoints

#### 3.3.1. AEs

Out of a total of 81 AEs, 79 were mild in severity, 1 was moderate, and 1 was severe. The most frequently reported AEs were nervous system disorders (29 out of 81) with an incidence of 66.7% in Placebo vs. 46.7% in the HB‐adMSC group, followed by general disorders such as fatigue, influenza‐like symptoms, and chills (15 out of 81), with an incidence of 44.4% in Placebo vs. 40% in the HB‐adMSC group and musculoskeletal and connective tissue disorders (10 out of 81) with an incidence of 55.6% in Placebo vs. 26.7% in the HB‐adMSC group. The majority of AEs (91.7%) were classified as unrelated to study treatment, and no serious AEs were considered related to HB‐adMSC administration (Table 3). For a detailed list of AEs and SAEs, see Supporting Information (Supporting Information Table S3). No deaths occurred during the entire treatment period. Overall, the majority of neurological AEs were assessed as unrelated to HB‐adMSC administration, and no pattern suggestive of treatment‐related neurological worsening was observed.

**TABLE 3:** Summary of adverse events by treatment group.

	**HB-adMSCs (*N* = 15)**		**Placebo (*N* = 9)**		**Overall (*N* = 24)**	
	***n* (%)**	**Events**	***n* (%)**	**Events**	***n* (%)**	**Events**

Adverse events	15 (100.0)	54	9 (100.0)	27	24 (100.0)	81
Nervous system disorders	7 (46.7)	19	6 (66.7)	10	13 (54.2)	29
General disorders and administration site conditions	6 (40.0)	11	4 (44.4)	4	10 (41.7)	15
Musculoskeletal and connective tissue disorders (for complete list of AEs, refer to Supporting Information)	4 (26.7)	4	5 (55.6)	6	9 (37.5)	10
Serious						
No	15 (100.0)	53	9 (100.0)	27	24 (100.0)	80
Yes	1 (6.7)	1	0 (0.0)	0	1 (4.2)	1
Severity						
Mild	15 (100.0)	52	9 (100.0)	27	24 (100.0)	79
Moderate	1 (6.7)	1	0 (0.0)	0	1 (4.2)	1
Severe	1 (6.7)	1	0 (0.0)	0	1 (4.2)	1
Relationship to study drug						
Possible	6 (40.0)	11	1 (11.1)	2	7 (29.2)	13
Probable	5 (33.3)	12	0 (0.0)	0	5 (20.8)	12
Unlikely	3 (20.0)	4	2 (22.2)	2	5 (20.8)	6
Unrelated	14 (93.3)	26	8 (88.9)	23	22 (91.7)	49
Not applicable	1 (6.7)	1	0 (0.0)	0	1 (4.2)	1
Action						
Drug withdrawn	0 (0.0)	0	0 (0.0)	0	0 (0.0)	0
Dose reduced	0 (0.0)	0	0 (0.0)	0	0 (0.0)	0
Dose increased	0 (0.0)	0	0 (0.0)	0	0 (0.0)	0
Dose not changed	13 (86.7)	40	8 (88.9)	12	21 (87.5)	52
Unknown	0 (0.0)	0	0 (0.0)	0	0 (0.0)	0
Not applicable	9 (60.0)	14	6 (66.7)	15	15 (62.5)	29
Outcome						
Fatal	0 (0.0)	0	0 (0.0)	0	0 (0.0)	0
Not recovered/not resolved	4 (26.7)	5	5 (55.6)	12	9 (37.5)	17
Recovered/resolved with sequelae	1 (6.7)	1	1 (11.1)	1	2 (8.3)	2
Recovering/resolving	1 (6.7)	1	2 (22.2)	4	3 (12.5)	5
Recovered/resolved	14 (93.3)	47	6 (66.7)	10	20 (83.3)	57
Unknown	0 (0.0)	0	0 (0.0)	0	0 (0.0)	0
Not applicable	0 (0.0)	0	0 (0.0)	0	0 (0.0)	0

*Note:* HB‐adMSCs, Hope Biosciences adipose‐derived MSCs (200 million). Please see Supporting Information for the full list of adverse events and serious adverse events, by each system organ class.

#### 3.3.2. Laboratory Evaluation

Comparison of the standard laboratory evaluations, including comprehensive metabolic panel (CMP), hematology, and coagulation panel at the baseline and EOS showed no clinically relevant differences between the two groups (Supporting Information Table S4). Also, no significant changes were observed in any of the laboratory parameters when compared with the baseline.

## 4. Discussion

The current study investigated the safety and preliminary efficacy of multiple intravenous infusions of autologous, HB‐adMSCs in improving signs and symptoms of patients with PD. Our findings indicate that the treatment regimen was safe and well tolerated, with no detrimental effects of the investigational product observed, as evidenced by standard laboratory measures, physical examinations, and vital signs.

Throughout the study period, only one SAE was reported that was determined to be unrelated to the investigational product. Importantly, the incidence of the most frequently reported AEs was higher in the placebo than in the HB‐adMSC group. This suggests that the treatment with HB‐adMSCs may offer a favorable safety profile and better tolerability, positioning it as a viable candidate for further research in PD therapy. These findings underscore the potential of HB‐adMSC therapy in the treatment of PD, emphasizing safety and tolerability as critical considerations for future clinical trials and therapeutic applications.

However, despite the favorable safety profile, the study’s findings on MDS‐UPDRS Part II and other efficacy measures should be interpreted with caution, as this early‐phase trial was primarily intended to evaluate safety and feasibility in a small cohort. No statistically significant improvements between the HB‐adMSC and the Placebo groups were observed, in the primary and secondary efficacy endpoints. The results may be influenced by several factors, including the small sample size, potential imbalance in randomization, and uneven distribution of Parkinsonian symptom severity, particularly affecting the HB‐adMSC group. Despite randomization was performed to prevent enrollment bias, an imbalance emerged between groups at baseline, with the HB‐adMSC group exhibiting more severe PD symptoms, thereby exposing these subjects to more vulnerable PD symptoms. For instance, the HB‐adMSC group exhibited higher baseline PDQ‐39 scores compared with the Placebo group, indicating that individuals in the treatment arm perceived a more pronounced impact of PD on their quality of life before the intervention began. This disparity highlights inherent differences in disease burden and subjective health status between groups, which may have masked potential treatment effects. Moreover, the MDS‐UPDRS II assessment, utilized for the primary endpoint, has documented limitations in reliability and psychometric properties [16], which could have compromised the precision of our evaluations.

Our designation of mild‐to‐moderate disease was intended to capture relative differences in symptom burden within this small cohort rather than to strictly follow conventional severity cutoffs. Recognizing this helps contextualize the observed stability in MDS‐UPDRS scores and the interpretation of efficacy outcomes, particularly given the predominantly mild disease profile of the enrolled participants.

Another factor to consider is the dosing regimen. The study used initial monthly infusions followed by bimonthly infusions, which may influence outcomes. In a previous single‐patient case study [17], monthly infusions appeared important for maintaining benefits and preventing symptom relapse. Similarly, Shigematsu et al. [18] reported improvements in three PD patients with roughly monthly MSC infusions. These observations suggest that infusion frequency could be an important consideration for future studies though this trial was primarily designed to assess safety and feasibility.

Despite the lack of statistically significant efficacy, an important finding from our study is that both the HB‐adMSC and the Placebo groups exhibited stability in MDS‐UPDRS Parts I, II, and III scores over the observed period. Neither group experienced symptom worsening, which is a critical outcome in PD management. The lack of symptom deterioration may reflect the benefits of the study’s therapeutic context, including enhanced clinical monitoring, regular patient care, and engagement, regardless of the intervention.

This trial aligns with previous research indicating that MSC therapies are safe and well tolerated, reinforcing the potential of MSC‐based therapies in treating neurodegenerative diseases like PD. [18–21]. The current study is among the few to investigate the safety and efficacy of multiple infusions of autologous adipose‐derived MSCs for PD treatment [18], highlighting the need for further research into their therapeutic efficacy and optimal administration protocols.

This trial is not without limitations. First, the sample size of this randomized, placebo‐controlled study was small, potentially impacting the study’s statistical power. No formal a priori sample size calculation or power analysis was performed, as the study was designed primarily as an early‐phase safety and feasibility trial rather than a confirmatory efficacy study. Second, despite randomization, there was an imbalance observed at baseline between group sizes (15 subjects in HB‐adMSC vs. 9 in Placebo group), which could have compromised the robustness of efficacy estimates. Third, unlike previous studies that utilized advanced imaging techniques for more comprehensive and objective assessments, our reliance on subjective measures may have introduced bias into the evaluation of treatment outcomes. Additionally, the absence of biomarker analyses limited our ability to explore the underlying biological effects of HB‐adMSC therapy, including potential impacts on nondopaminergic pathways implicated in PD. PD is increasingly recognized as a multisystem disorder involving not only dopaminergic degeneration but also nondopaminergic neurodegeneration, including cholinergic, noradrenergic, and serotonergic systems [22]. In particular, cholinergic dysfunction has been associated with nonmotor symptoms such as cognitive decline, gait disturbances, and postural instability, which are often resistant to standard dopaminergic treatments [23–26].

A further limitation relates to the use of autologous adipose‐derived MSCs. Although adMSCs generally exhibit low immunogenicity and strong immunomodulatory properties, cells from individuals with PD may harbor mitochondrial dysfunction, impaired proteostasis, or increased oxidative stress, which could affect their trophic or reparative capacity. These factors, combined with inherent donor‐to‐donor variability, may influence cell quality across participants. Importantly, despite these potential challenges, the validated, cGMP‐compliant manufacturing process preserves MSC identity, genetic stability, and functional potency throughout expansion. In this early, safety‐focused trial, we chose autologous adMSCs to minimize immunologic risk and enhance feasibility, but these factors should be considered when interpreting the findings.

Another limitation pertains to clinical assessments. MDS‐UPDRS evaluations were conducted by clinicians trained and certified by the International Parkinson and Movement Disorder Society, using standardized procedures to support reliability. Nevertheless, a small degree of diagnostic or rating variability cannot be excluded, particularly in a study of limited sample size, and the results should, therefore, be interpreted with caution. All assessments were performed in the ON‐medication state. While OFF‐state evaluations could provide additional insight into underlying motor severity and may detect subtle treatment effects, they were not included in this small, early‐phase trial due to day‐to‐day symptom variability, subjective differences in patient‐reported OFF episodes, nonmotor contributions, and the added burden of medication withdrawal for participants. As a result, all clinical assessments were conducted in the ON‐medication state, which may have reduced sensitivity to detect treatment effects, although future studies could consider standardized OFF‐state evaluations to more precisely assess treatment effects.

Although this trial did not incorporate neuroimaging or mechanistic assays, in our prior FDA‐authorized single‐patient Expanded Access IND study using HB‐adMSCs, we performed longitudinal FDG‐PET imaging and observed region‐specific changes in brain glucose metabolism following treatment [17]. While FDG‐PET does not directly measure cholinergic function, these metabolic changes may reflect modulation of broader neural networks, potentially mediated by MSC‐secreted factors that influence neuroinflammation and neuronal support pathways. Given the involvement of cholinergic pathways in the pathophysiology of nonmotor symptoms in PD, future studies should consider incorporating early postinfusion biomarker assessments (e.g., cholinesterase activity) and neurotransmitter‐specific imaging to investigate whether MSC therapies exert modulatory effects on cholinergic and other nondopaminergic pathways. Although speculative, such exploration could provide insights into broader mechanisms of MSC action beyond conventional dopaminergic targets.

While the precise mechanisms of MSC action in PD remain under investigation, current evidence supports a central role for peripheral paracrine and immunomodulatory effects. Several preclinical and clinical studies suggest that MSCs can exert biologically meaningful effects on the central nervous system without requiring direct CNS engraftment. These effects are mediated through the secretion of anti‐inflammatory cytokines, neurotrophic factors (e.g., BDNF, NGF, and VEGF), and extracellular vesicles (EVs), which modulate systemic immune responses and can indirectly influence neuroinflammation, neuronal survival, and functional stability [27–30]. Through these mechanisms, MSC therapy may promote neuronal support, reduce neuroinflammation, and stabilize motor and nonmotor symptoms in PD. These mechanistic insights provide a biologically plausible rationale for intravenous MSC therapy in PD, explaining how repeated systemic infusions could modulate neuroinflammation and support neuronal function, independent of direct CNS localization.

## 5. Conclusions

In this small, early‐phase PD trial, multiple infusions of 200 million autologous HB‐adMSCs were safe and well tolerated, with predominantly mild AEs reported. Patient‐reported outcomes, including MDS‐UPDRS Part II in the ON‐medication state, demonstrated that repeated infusions are feasible and generally well accepted by participants, even in early‐stage PD. While the study was not powered to provide definitive efficacy conclusions and the cohort was small, these findings provide foundational safety and feasibility data. The results support further exploration in larger, balanced trials with longer follow‐up to better characterize the potential therapeutic effects of HB‐adMSCs in PD.

## Author Contributions

Ridhima Vij: formal analysis, roles/writing–original draft, and writing–review and editing; Hosu Kim: resources; Hyeonggeun Park: resources; Thanh Cheng: data curation, investigation, supervision, and methodology; Djamchid Lotfi: data curation, investigation, supervision, and methodology; Donna Chang: conceptualization and project administration, funding acquisition, and writing–review and editing.

## Funding

No funding was received for this research.

## Conflicts of Interest

Hosu Kim, Hyeonggeun Park, and Donna Chang are shareholders of Hope Biosciences LLC, which may be perceived as a potential conflicts of interest. The remaining authors declare no conflicts of interest.

## Supporting Information

Additional supporting information can be found online in the Supporting Information section.

## Supporting information


**Supporting Information** Table S1 includes MSC quality control metrics for all six infusions for *N* = 15 subjects, who received infusions with 200 million HB‐adMSCs. Table S2 provides a summary of medical history for all 24 subjects. Table S3 summarizes all adverse events and serious adverse events by system organ class. Table S4 provides details of the laboratory parameters including the comprehensive metabolic panel, hematology, and coagulation, both at baseline and at the end of the study.

## Data Availability

The data that support the findings of this study are available on request from the corresponding author.
