# Supplementary material for: Evaluation of Multiple Intravenous Infusions of Autologous Adipose‐Derived Mesenchymal Stem Cells in Parkinson’s Disease: A Randomized, Double‐Blind Clinical Trial
Source: Parkinsons Dis. 2026 May 13;2026:9934417. doi: 10.1155/padi/9934417 (PMC13168863; doi:10.1155/padi/9934417)
Supplement: Supplementary file 1 — Supporting Information Table S1 includes MSC quality control metrics for all six infusions for N = 15 subjects, who received infusions with 200 million HB‐adMSCs. Table S2 provides a summary of medical history for all 24 subjects. Table S3 summarizes all adverse events and serious adverse events by system organ class. Table S4 provides details of the laboratory parameters including the comprehensive metabolic panel, hematology, and coagulation, both at baseline and at the end of the study. [file PADI-2026-9934417-s001.docx]

Table S1. MSC quality control metrics for all six infusions (N=15 subjects, who received infusions with 200 MM HB-adMSCs)

| **Subject #** | **Infusion #** | **Total cell count (million)** | **Cell Viability (%)** | **CD73 (%)** | **CD29 (%)** | **CD31 (%)** | **CD45 (%)** |
| --- | --- | --- | --- | --- | --- | --- | --- |
| HB-adMSC#1 | 1 | 240 | 98.11 | 94.51 | 98.8 | 0 | 0.26 |
|  | 2 | 214 | 94.37 | 99.55 | 99.9 | 0 | 0.25 |
|  | 3 | 240 | 95.06 | 94.79 | 99.82 | 0 | 0.22 |
|  | 4 | 240 | 94.05 | 87.44 | 99.97 | 0 | 0 |
|  | 5 | 221 | 97.18 | 92.41 | 99.96 | 0 | 0.36 |
|  | 6 | 240 | 97.47 | 97.84 | 100 | 0 | 0.12 |
| HB-adMSC#2 | 1 | 84.8* | 100.00 | 97.89 | 99.51 | 0 | 0 |
|  | 2 | 240 | 96.81 | 99.89 | 100 | 0 | 0.16 |
|  | 3 | 240 | 94.44 | 99.29 | 99.89 | 0 | 0.22 |
|  | 4 | 218 | 97.14 | 87.79 | 99.62 | 0 | 0 |
|  | 5 | 240 | 98.56 | 95.85 | 99.94 | 0 | 0.38 |
|  | 6 | 240 | 97.75 | 98.88 | 99.95 | 0.05 | 0.54 |
| HB-adMSC#3 | 1 | 173 | 94.74 | 98.03 | 99.84 | 0 | 0.1 |
|  | 2 | 166 | 96.30 | 89.64 | 99.95 | 0 | 0.25 |
|  | 3 | 240 | 97.54 | 86.94 | 99.85 | 0 | 0.1 |
|  | 4 | 240 | 98.17 | 97.62 | 99.63 | 0 | 0.19 |
|  | 5 | 230 | 97.30 | 98.24 | 99.17 | 0 | 0.22 |
|  | 6 | 240 | 96.77 | 92.25 | 99.92 | 0 | 0.78 |
| HB-adMSC#4 | 1 | 189 | 96.72 | 99.36 | 99.94 | 0 | 0.12 |
|  | 2 | 141 | 97.78 | 87.64 | 99.51 | 0 | 0.27 |
|  | 3 | 237 | 98.84 | 90.2 | 99.9 | 0 | 0.05 |
|  | 4 | 189 | 98.33 | 98.17 | 99.73 | 0 | 0.48 |
|  | 5 | 192 | 96.77 | 97.6 | 98.35 | 0 | 0.63 |
|  | 6 | 240 | 97.83 | 93.33 | 99.91 | 0.09 | 1.46 |
| HB-adMSC#5 | 1 | 224 | 97.22 | 93.76 | 100 | 0 | 0.35 |
|  | 2 | 240 | 97.00 | 94.37 | 99.87 | 0 | 0.03 |
|  | 3 | 240 | 96.35 | 98.3 | 99.48 | 0.04 | 0.31 |
|  | 4 | 240 | 98.68 | 82.91 | 99.84 | 0 | 0.17 |
|  | 5 | 214 | 97.10 | 79.67 | 99.95 | 0 | 0.2 |
|  | 6 | 240 | 96.30 | 91.11 | 100 | 0 | 0.13 |
| HB-adMSC#6 | 1 | 202 | 98.44 | 87.09 | 99.42 | 0 | 0.26 |
|  | 2 | 208 | 92.86 | 92 | 99.85 | 0.04 | 0.07 |
|  | 3 | 240 | 96.55 | 97.54 | 99.02 | 0.41 | 0.57 |
|  | 4 | 234 | 97.33 | 83.22 | 99.79 | 0.05 | 0.16 |
|  | 5 | 234 | 96.05 | 77.28 | 99.82 | 0 | 0.36 |
|  | 6 | 240 | 97.03 | 88.89 | 99.96 | 0 | 0.22 |
| HB-adMSC#7 | 1 | 240 | 97.22 | 98.4 | 99.63 | 0 | 1.01 |
|  | 2 | 240 | 96.15 | 86.7 | 100 | 0.07 | 0.22 |
|  | 3 | 240 | 94.79 | 91.76 | 99.93 | 0 | 0.07 |
|  | 4 | 214 | 98.53 | 93.37 | 100 | 0 | 0.21 |
|  | 5 | 240 | 98.86 | 93.95 | 100 | 0 | 0.05 |
|  | 6 | 160 | 96.15 | 98 | 99.94 | 0 | 0.61 |
| HB-adMSC#8 | 1 | 230 | 97.30 | 98.78 | 99.9 | 0.05 | 0.46 |
|  | 2 | 224 | 98.59 | 96.25 | 100 | 0 | 0.55 |
|  | 3 | 123** | 95.65 | 89.96 | 99.93 | 0 | 0 |
|  | 4 | 240 | 97.56 | 87.53 | 99.94 | 0 | 0.06 |
|  | 5 | 195 | 96.83 | 96.53 | 99.8 | 0 | 0.15 |
|  | 6 | 214 | 97.10 | 97.83 | 99.83 | 0 | 0.84 |
| HB-adMSC#9 | 1 | 170 | 94.64 | 98.75 | 99.75 | 0 | 0.33 |
|  | 2 | 198 | 95.38 | 93.38 | 100 | 0 | 0.35 |
|  | 3 | 141*** | 97.78 | 93.42 | 99.74 | 0 | 0.37 |
|  | 4 | 176 | 96.49 | 86.86 | 100 | 0 | 0.22 |
|  | 5 | 227 | 93.42 | 85.37 | 99.94 | 0 | 0.13 |
|  | 6 | 211 | 97.06 | 98.57 | 99.81 | 0 | 0.05 |
| HB-adMSC#10 | 1 | 221 | 94.52 | 97.85 | 99.98 | 0.02 | 0.29 |
|  | 2 | 240 | 93.83 | 93.83 | 99.97 | 0 | 0.45 |
|  | 3 | 234 | 97.33 | 93.57 | 99.34 | 0 | 0.29 |
|  | 4 | 240 | 97.47 | 94.12 | 99.95 | 0.05 | 0.1 |
|  | 5 | 240 | 96.30 | 81.84 | 100 | 0 | 0.11 |
|  | 6 | 240 | 96.00 | 99.47 | 100 | 0 | 0.49 |
| HB-adMSC#11 | 1 | 240 | 99.27 | 88.46 | 99.69 | 0 | 0.49 |
|  | 2 | 192 | 96.77 | 98.33 | 99.78 | 0 | 0 |
|  | 3 | 240 | 98.75 | 86.93 | 99.93 | 0 | 0.5 |
|  | 4 | 240 | 98.88 | 96.62 | 99.95 | 0 | 0.46 |
|  | 5 | 227 | 95.95 | 98.62 | 100 | 0 | 0.52 |
|  | 6 | 214 | 98.53 | 88.45 | 93.77 | 0.06 | 0.4 |
| HB-adMSC#12 | 1 | 240 | 95.41 | 84.29 | 99.79 | 0.04 | 0.55 |
|  | 2 | 173 | 98.18 | 84.94 | 99.91 | 0 | 0 |
|  | 3 | 240 | 98.92 | 86.23 | 99.86 | 0.05 | 0.67 |
|  | 4 | 224 | 94.59 | 96.44 | 99.95 | 0 | 0.55 |
|  | 5 | 218 | 97.14 | 98.36 | 99.88 | 0 | 0.45 |
|  | 6 | 237 | 92.50 | 94.45 | 99.34 | 0 | 0.26 |
| HB-adMSC#13 | 1 | 205 | 100.00 | 85.75 | 99.96 | 0 | 0.38 |
|  | 2 | 230 | 98.63 | 87.82 | 99.89 | 0 | 0.04 |
|  | 3 | 176 | 93.22 | 95.08 | 99.9 | 0 | 0.53 |
|  | 4 | 181 | 97.41 | 96.24 | 100 | 0.04 | 0.28 |
|  | 5 | 170 | 96.36 | 95.23 | 99.96 | 0 | 0.26 |
|  | 6 | 211 | 98.51 | 95.81 | 99.08 | 0 | 0.23 |
| HB-adMSC#14 | 1 | 240 | 96.74 | 89.1 | 99.8 | 0 | 0.33 |
|  | 2 | 240 | 98.25 | 99.31 | 99.74 | 0 | 0.3 |
|  | 3 | 240 | 100.00 | 95.69 | 99.95 | 0 | 0.7 |
|  | 4 | 195 | 96.83 | 95.63 | 100 | 0 | 0.11 |
|  | 5 | 240 | 95.70 | 97.51 | 100 | 0 | 0.29 |
|  | 6 | 240 | 98.99 | 98.65 | 99.58 | 0 | 0.33 |
| HB-adMSC#15 | 1 | 195 | 95.31 | 79.91 | 100 | 0 | 0.47 |
|  | 2 | 240 | 96.59 | 97.87 | 100 | 0 | 0.68 |
|  | 3 | 240 | 97.90 | 90.67 | 100 | 0 | 0.11 |
|  | 4 | 240 | 98.84 | 98.25 | 99.86 | 0 | 1.23 |
|  | 5 | 234 | 94.81 | 97.81 | 99.8 | 0 | 0.41 |
|  | 6 | 240 | 97.59 | 100 | 100 | 0 | 0 |

*Scheduling error, which allowed for only 9 days of production period until release

**Cell loss due to re-filtration performed to remove cell clumps after initial syringe filling

***Donor variability in the yield being low per unit surface area of culture flask. The products for the following infusions were produced using more flasks

**Table S2. Summary of medical history for all (N=24) subjects**

|  | | **HB-adMSCs (N=15)** | | **Placebo (N=9)** | | **Overall (N=24)** | |
| --- | --- | --- | --- | --- | --- | --- | --- |
|  | | **n (%)** | | **n (%)** | | **n (%)** | |
| Subjects with Medical History | | 15 (100.0) | | 9 (100.0) | | 24 (100.0) | |
|  | | | | | | | |
| Parkinson's disease | | 15 (100.0) | | 9 (100.0) | | 24 (100.0) | |
| Administration of influenza vaccine | | 7 (46.7) | | 3 (33.3) | | 10 (41.7) | |
| COVID-19 vaccine | | 6 (40.0) | | 4 (44.4) | | 10 (41.7) | |
| Administration of Varicella-zoster vaccine for shingles | | 4 (26.7) | | 2 (22.2) | | 6 (25.0) | |
| Hypertensive disorder | | 4 (26.7) | | 2 (22.2) | | 6 (25.0) | |
| Snoring | | 3 (20.0) | | 3 (33.3) | | 6 (25.0) | |
| Hypercholesterolemia | | 2 (13.3) | | 3 (33.3) | | 5 (20.8) | |
| Osteoarthritis | | 2 (13.3) | | 3 (33.3) | | 5 (20.8) | |
| Administration of hepatitis A vaccine | | 1 (6.7) | | 3 (33.3) | | 4 (16.7) | |
| Administration of hepatitis B vaccine | | 1 (6.7) | | 3 (33.3) | | 4 (16.7) | |
| Anxiety | | 3 (20.0) | | 1 (11.1) | | 4 (16.7) | |
| Seasonal allergy | | 2 (13.3) | | 2 (22.2) | | 4 (16.7) | |
| Tonsillectomy | | 4 (26.7) | | 0 (0.0) | | 4 (16.7) | |
| Administration of pneumococcal vaccine | | 3 (20.0) | | 0 (0.0) | | 3 (12.5) | |
| Administration of tetanus vaccine | | 2 (13.3) | | 1 (11.1) | | 3 (12.5) | |
| Benign prostatic hyperplasia | | 3 (20.0) | | 0 (0.0) | | 3 (12.5) | |
| Chronic constipation | | 1 (6.7) | | 2 (22.2) | | 3 (12.5) | |
| Decreased testosterone level | | 1 (6.7) | | 2 (22.2) | | 3 (12.5) | |
| Loss of hair | | 1 (6.7) | | 2 (22.2) | | 3 (12.5) | |
| Low back pain | | 2 (13.3) | | 1 (11.1) | | 3 (12.5) | |
| Menopause | | 2 (13.3) | | 1 (11.1) | | 3 (12.5) | |
| Polyp of colon | | 1 (6.7) | | 2 (22.2) | | 3 (12.5) | |
| Tetanus, diphtheria, and acellular pertussis vaccination | | 3 (20.0) | | 0 (0.0) | | 3 (12.5) | |
| Trachyonychia | | 1 (6.7) | | 2 (22.2) | | 3 (12.5) | |
| Appendectomy | | 1 (6.7) | | 1 (11.1) | | 2 (8.3) | |
| Cesarean delivery - delivered | | 2 (13.3) | | 0 (0.0) | | 2 (8.3) | |
| Extraction of wisdom tooth | | 1 (6.7) | | 1 (11.1) | | 2 (8.3) | |
| Gastroesophageal reflux disease | | 2 (13.3) | | 0 (0.0) | | 2 (8.3) | |
| Glaucoma | | 2 (13.3) | | 0 (0.0) | | 2 (8.3) | |
| Hypothyroidism | | 2 (13.3) | | 0 (0.0) | | 2 (8.3) | |
| Kidney stone | | 0 (0.0) | | 2 (22.2) | | 2 (8.3) | |
| Neuropathy | | 1 (6.7) | | 1 (11.1) | | 2 (8.3) | |
| Psoriasis | | 2 (13.3) | | 0 (0.0) | | 2 (8.3) | |
| Sleep apnea | | 1 (6.7) | | 1 (11.1) | | 2 (8.3) | |
| Tubal ligation done | | 2 (13.3) | | 0 (0.0) | | 2 (8.3) | |
| Tuberculosis | | 1 (6.7) | | 1 (11.1) | | 2 (8.3) | |
| Abnormal heartbeat | | 1 (6.7) | | 0 (0.0) | | 1 (4.2) | |
| Administration of SARS-CoV-2 mRNA vaccine | | 0 (0.0) | | 1 (11.1) | | 1 (4.2) | |
| Administration of booster dose of tetanus vaccine | | 0 (0.0) | | 1 (11.1) | | 1 (4.2) | |
| Administration of first dose of SARS-CoV-2 mRNA vaccine | | 1 (6.7) | | 0 (0.0) | | 1 (4.2) | |
| Administration of second dose of SARS-CoV-2 mRNA vaccine | | 1 (6.7) | | 0 (0.0) | | 1 (4.2) | |
| Allergic rhinitis | | 0 (0.0) | | 1 (11.1) | | 1 (4.2) | |
| Allergies reference set for GP/FP health issue | | 1 (6.7) | | 0 (0.0) | | 1 (4.2) | |
| Alopecia | | 0 (0.0) | | 1 (11.1) | | 1 (4.2) | |
| Anti-phospholipid antibody | | 1 (6.7) | | 0 (0.0) | | 1 (4.2) | |
| Arthritis | | 1 (6.7) | | 0 (0.0) | | 1 (4.2) | |
| Arthroscopic knee operation | | 1 (6.7) | | 0 (0.0) | | 1 (4.2) | |
| Arthroscopy of knee with lateral meniscus repair | | 1 (6.7) | | 0 (0.0) | | 1 (4.2) | |
| Attention deficit hyperactivity disorder | | 1 (6.7) | | 0 (0.0) | | 1 (4.2) | |
| Autoimmune hypothyroidism | | 1 (6.7) | | 0 (0.0) | | 1 (4.2) | |
| Barrett's esophagus | | 0 (0.0) | | 1 (11.1) | | 1 (4.2) | |
| Bilateral cataracts | | 0 (0.0) | | 1 (11.1) | | 1 (4.2) | |
| Bilateral extraction of cataracts | | 0 (0.0) | | 1 (11.1) | | 1 (4.2) | |
| Bilateral obstruction of fallopian tubes | | 0 (0.0) | | 1 (11.1) | | 1 (4.2) | |
| Bilateral spontaneous rupture of tympanic membranes of ears co-occurrent and due to recurrent acute suppurative otitis media | | 0 (0.0) | | 1 (11.1) | | 1 (4.2) | |
| Blepharoplasty | | 0 (0.0) | | 1 (11.1) | | 1 (4.2) | |
| Blurring of visual image | | 1 (6.7) | | 0 (0.0) | | 1 (4.2) | |
| Bunion | | 1 (6.7) | | 0 (0.0) | | 1 (4.2) | |
| Bypass of segment of aorta | | 1 (6.7) | | 0 (0.0) | | 1 (4.2) | |
| COVID-19 | | 0 (0.0) | | 1 (11.1) | | 1 (4.2) | |
| Calculus | | 1 (6.7) | | 0 (0.0) | | 1 (4.2) | |
| Cerebrovascular accident | | 0 (0.0) | | 1 (11.1) | | 1 (4.2) | |
| Colonoscopy | | 0 (0.0) | | 1 (11.1) | | 1 (4.2) | |
| Congenital prolapse of bladder | | 1 (6.7) | | 0 (0.0) | | 1 (4.2) | |
| Constipation | | 1 (6.7) | | 0 (0.0) | | 1 (4.2) | |
| Coronavirus infection | | 1 (6.7) | | 0 (0.0) | | 1 (4.2) | |
| Decompression of spinal cord | | 1 (6.7) | | 0 (0.0) | | 1 (4.2) | |
| Dilation and curettage of uterus after delivery | | 0 (0.0) | | 1 (11.1) | | 1 (4.2) | |
| Dilation of cervix uteri and curettage of products of conception from uterus | | 0 (0.0) | | 1 (11.1) | | 1 (4.2) | |
| Discectomy of spine | | 0 (0.0) | | 1 (11.1) | | 1 (4.2) | |
| Displacement of breast implant | | 1 (6.7) | | 0 (0.0) | | 1 (4.2) | |
| ECG: ectopic beats - premature atrial contraction | | 0 (0.0) | | 1 (11.1) | | 1 (4.2) | |
| Elevated blood pressure | | 1 (6.7) | | 0 (0.0) | | 1 (4.2) | |
| Elevated cholesterol/high density lipoprotein ratio | | 1 (6.7) | | 0 (0.0) | | 1 (4.2) | |
| Endometrial ablation | | 1 (6.7) | | 0 (0.0) | | 1 (4.2) | |
| Erectile dysfunction | | 0 (0.0) | | 1 (11.1) | | 1 (4.2) | |
| Extracorporeal shockwave lithotripsy of calculus of kidney | | 1 (6.7) | | 0 (0.0) | | 1 (4.2) | |
| Facelift and tightening of platysma | | 0 (0.0) | | 1 (11.1) | | 1 (4.2) | |
| Facial rhytidoplasty | | 0 (0.0) | | 1 (11.1) | | 1 (4.2) | |
| Floaters in visual field | | 0 (0.0) | | 1 (11.1) | | 1 (4.2) | |
| Fracture of bone | | 1 (6.7) | | 0 (0.0) | | 1 (4.2) | |
| Fracture of olecranon | | 0 (0.0) | | 1 (11.1) | | 1 (4.2) | |
| Fracture of upper limb | | 0 (0.0) | | 1 (11.1) | | 1 (4.2) | |
| Genital warts | | 0 (0.0) | | 1 (11.1) | | 1 (4.2) | |
| Gout | | 0 (0.0) | | 1 (11.1) | | 1 (4.2) | |
| Hammer toe | | 1 (6.7) | | 0 (0.0) | | 1 (4.2) | |
| Hand tendon repaired | | 1 (6.7) | | 0 (0.0) | | 1 (4.2) | |
| Heart murmur | | 1 (6.7) | | 0 (0.0) | | 1 (4.2) | |
| Hemorrhoids | | 1 (6.7) | | 0 (0.0) | | 1 (4.2) | |
| Hernia repair | | 1 (6.7) | | 0 (0.0) | | 1 (4.2) | |
| History of eyelid surgery | | 1 (6.7) | | 0 (0.0) | | 1 (4.2) | |
| History of hepatitis B vaccination | | 0 (0.0) | | 1 (11.1) | | 1 (4.2) | |
| History of influenza vaccination | | 0 (0.0) | | 1 (11.1) | | 1 (4.2) | |
| History of partial resection of colon | | 1 (6.7) | | 0 (0.0) | | 1 (4.2) | |
| History of polyp of colon | | 0 (0.0) | | 1 (11.1) | | 1 (4.2) | |
| History of right hip replacement | | 1 (6.7) | | 0 (0.0) | | 1 (4.2) | |
| Hypothyroidism due to Hashimoto's thyroiditis | | 1 (6.7) | | 0 (0.0) | | 1 (4.2) | |
| Implantation of joint prosthesis | | 0 (0.0) | | 1 (11.1) | | 1 (4.2) | |
| Incision of colon and excision of polyp | | 1 (6.7) | | 0 (0.0) | | 1 (4.2) | |
| Incision of uvula | | 1 (6.7) | | 0 (0.0) | | 1 (4.2) | |
| Injection of frozen shoulder | | 0 (0.0) | | 1 (11.1) | | 1 (4.2) | |
| Insertion of bilateral silicone gel-filled breast implants | | 1 (6.7) | | 0 (0.0) | | 1 (4.2) | |
| Iridocorneal endothelial syndrome | | 1 (6.7) | | 0 (0.0) | | 1 (4.2) | |
| Irregular periods | | 1 (6.7) | | 0 (0.0) | | 1 (4.2) | |
| Joint pain | | 0 (0.0) | | 1 (11.1) | | 1 (4.2) | |
| Loss of taste | | 1 (6.7) | | 0 (0.0) | | 1 (4.2) | |
| Lumbosacral radiculoplexus neuropathy due to type 2 diabetes mellitus | | 1 (6.7) | | 0 (0.0) | | 1 (4.2) | |
| Male pattern alopecia | | 1 (6.7) | | 0 (0.0) | | 1 (4.2) | |
| Malignant tumor of colon | | 1 (6.7) | | 0 (0.0) | | 1 (4.2) | |
| Melanoma in situ of scalp | | 0 (0.0) | | 1 (11.1) | | 1 (4.2) | |
| Microlaryngoscopy | | 1 (6.7) | | 0 (0.0) | | 1 (4.2) | |
| Mucosal anosmia | | 1 (6.7) | | 0 (0.0) | | 1 (4.2) | |
| Neck pain | | 1 (6.7) | | 0 (0.0) | | 1 (4.2) | |
| Night sweats | | 0 (0.0) | | 1 (11.1) | | 1 (4.2) | |
| Obesity | | 1 (6.7) | | 0 (0.0) | | 1 (4.2) | |
| Open heart surgery | | 1 (6.7) | | 0 (0.0) | | 1 (4.2) | |
| Operation on facial joint | | 1 (6.7) | | 0 (0.0) | | 1 (4.2) | |
| Osteoarthritis of hip | | 1 (6.7) | | 0 (0.0) | | 1 (4.2) | |
| Palpitations | | 1 (6.7) | | 0 (0.0) | | 1 (4.2) | |
| Parvovirus infection | | 0 (0.0) | | 1 (11.1) | | 1 (4.2) | |
| Pneumococcal conjugate vaccination | | 0 (0.0) | | 1 (11.1) | | 1 (4.2) | |
| Pneumonia | | 1 (6.7) | | 0 (0.0) | | 1 (4.2) | |
| Polyp | | 1 (6.7) | | 0 (0.0) | | 1 (4.2) | |
| Posterolateral lymph nodes neck dissection | | 1 (6.7) | | 0 (0.0) | | 1 (4.2) | |
| Raynaud's disease | | 1 (6.7) | | 0 (0.0) | | 1 (4.2) | |
| Repair of tendo achilles | | 1 (6.7) | | 0 (0.0) | | 1 (4.2) | |
| SARS coronavirus | | 0 (0.0) | | 1 (11.1) | | 1 (4.2) | |
| Saline-filled breast implant | | 1 (6.7) | | 0 (0.0) | | 1 (4.2) | |
| Sleep disorder | | 1 (6.7) | | 0 (0.0) | | 1 (4.2) | |
| Spinal stenosis in cervical region | | 1 (6.7) | | 0 (0.0) | | 1 (4.2) | |
| Stroke of uncertain pathology | | 0 (0.0) | | 1 (11.1) | | 1 (4.2) | |
| Surgical procedure on eye proper using laser | | 1 (6.7) | | 0 (0.0) | | 1 (4.2) | |
| Tendon strain | | 1 (6.7) | | 0 (0.0) | | 1 (4.2) | |
| Tremor | | 0 (0.0) | | 1 (11.1) | | 1 (4.2) | |
| Type 2 diabetes mellitus | | 1 (6.7) | | 0 (0.0) | | 1 (4.2) | |
| Uterine leiomyoma | | 1 (6.7) | | 0 (0.0) | | 1 (4.2) | |
| Vitiligo | | 1 (6.7) | | 0 (0.0) | | 1 (4.2) | |

| Table S3. Adverse Events and Serious Adverse Events by System Organ Class | | | | | | | | | | |  |
| --- | --- | --- | --- | --- | --- | --- | --- | --- | --- | --- | --- |
|  | | **HB-adMSCs (N=15)** | | | | | **Placebo (N=9)** | | **Overall (N=24)** | |  |
|  | | **n (%)** | | | | **Events** | **n (%)** | **Events** | **n (%)** | **Events** |  |
| **Adverse events** | | 15 (100.0) | | | | 54 | 9 (100.0) | 27 | 24 (100.0) | 81 |  |
|  | | | | | | | | | | |  |
| Nervous system disorders | | 7 (46.7) | | | | 19 | 6 (66.7) | 10 | 13 (54.2) | 29 |  |
| Headache | | 5 (33.3) | | | | 11 | 1 (11.1) | 1 | 6 (25.0) | 12 |  |
| Tremor | | 1 (6.7) | | | | 1 | 4 (44.4) | 6 | 5 (20.8) | 7 |  |
| Balance difficulty | | 0 (0.0) | | | | 0 | 1 (11.1) | 1 | 1 (4.2) | 1 |  |
| Dizziness | | 1 (6.7) | | | | 3 | 0 (0.0) | 0 | 1 (4.2) | 3 |  |
| Dystonia | | 1 (6.7) | | | | 1 | 0 (0.0) | 0 | 1 (4.2) | 1 |  |
| Frozen gait | | 0 (0.0) | | | | 0 | 1 (11.1) | 1 | 1 (4.2) | 1 |  |
| Migraine | | 1 (6.7) | | | | 1 | 0 (0.0) | 0 | 1 (4.2) | 1 |  |
| Near syncope | | 0 (0.0) | | | | 0 | 1 (11.1) | 1 | 1 (4.2) | 1 |  |
| Neuralgia | | 1 (6.7) | | | | 1 | 0 (0.0) | 0 | 1 (4.2) | 1 |  |
| Sedation | | 1 (6.7) | | | | 1 | 0 (0.0) | 0 | 1 (4.2) | 1 |  |
|  | | | | | | | | | | |  |
| General disorders and administration site conditions | | 6 (40.0) | | | | 11 | 4 (44.4) | 4 | 10 (41.7) | 15 |  |
| Fatigue | | 4 (26.7) | | | | 5 | 1 (11.1) | 1 | 5 (20.8) | 6 |  |
| Influenza-like symptoms | | 2 (13.3) | | | | 3 | 1 (11.1) | 1 | 3 (12.5) | 4 |  |
| Ankle edema | | 0 (0.0) | | | | 0 | 1 (11.1) | 1 | 1 (4.2) | 1 |  |
| Chills | | 1 (6.7) | | | | 1 | 0 (0.0) | 0 | 1 (4.2) | 1 |  |
| Drug ineffective | | 0 (0.0) | | | | 0 | 1 (11.1) | 1 | 1 (4.2) | 1 |  |
| Feverish | | 1 (6.7) | | | | 2 | 0 (0.0) | 0 | 1 (4.2) | 2 |  |
|  | | | | | | | | | | |  |
| Musculoskeletal and connective tissue disorders | | 4 (26.7) | | | | 4 | 5 (55.6) | 6 | 9 (37.5) | 10 |  |
| Muscle rigidity | | 2 (13.3) | | | | 2 | 3 (33.3) | 3 | 5 (20.8) | 5 |  |
| Joint pain | | 1 (6.7) | | | | 1 | 0 (0.0) | 0 | 1 (4.2) | 1 |  |
| Limb stiffness | | 0 (0.0) | | | | 0 | 1 (11.1) | 1 | 1 (4.2) | 1 |  |
| Spasms | | 1 (6.7) | | | | 1 | 0 (0.0) | 0 | 1 (4.2) | 1 |  |
| Stiff neck | | 0 (0.0) | | | | 0 | 1 (11.1) | 1 | 1 (4.2) | 1 |  |
| Swollen ankles | | 0 (0.0) | | | | 0 | 1 (11.1) | 1 | 1 (4.2) | 1 |  |
|  | | | | | | | | | | |  |
| Eye disorders | | 4 (26.7) | | | | 5 | 0 (0.0) | 0 | 4 (16.7) | 5 |  |
| Hazy vision | | 2 (13.3) | | | | 2 | 0 (0.0) | 0 | 2 (8.3) | 2 |  |
| Cataract | | 1 (6.7) | | | | 1 | 0 (0.0) | 0 | 1 (4.2) | 1 |  |
| Eyelid twitching | | 1 (6.7) | | | | 1 | 0 (0.0) | 0 | 1 (4.2) | 1 |  |
| Eyes heavy feeling of | | 1 (6.7) | | | | 1 | 0 (0.0) | 0 | 1 (4.2) | 1 |  |
|  | | | | | | | | | | |  |
| Infections and infestations | | 3 (20.0) | | | | 3 | 0 (0.0) | 0 | 3 (12.5) | 3 |  |
| COVID-19 | | 3 (20.0) | | | | 3 | 0 (0.0) | 0 | 3 (12.5) | 3 |  |
|  | | | | | | | | | | |  |
| Injury, poisoning and procedural complications | | 2 (13.3) | | | | 3 | 1 (11.1) | 1 | 3 (12.5) | 4 |  |
| Fall | | 2 (13.3) | | | | 3 | 0 (0.0) | 0 | 2 (8.3) | 3 |  |
| Muscle strain | | 0 (0.0) | | | | 0 | 1 (11.1) | 1 | 1 (4.2) | 1 |  |
|  | | | | | | | | | | |  |
| Psychiatric disorders | | 2 (13.3) | | | | 2 | | 1 (11.1) | 1 | 3 (12.5) | 3 |
| Insomnia | | 0 (0.0) | | | | 0 | | 1 (11.1) | 1 | 1 (4.2) | 1 |
| Mixed anxiety and depressive disorder | | 1 (6.7) | | | | 1 | | 0 (0.0) | 0 | 1 (4.2) | 1 |
| Suicidal ideation | | 1 (6.7) | | | | 1 | | 0 (0.0) | 0 | 1 (4.2) | 1 |
|  | | | | | | | | | | | |
| Skin and subcutaneous tissue disorders | | 1 (6.7) | | | | 1 | | 1 (11.1) | 1 | 2 (8.3) | 2 |
| Night sweats | | 0 (0.0) | | | | 0 | | 1 (11.1) | 1 | 1 (4.2) | 1 |
| Psoriasis | | 1 (6.7) | | | | 1 | | 0 (0.0) | 0 | 1 (4.2) | 1 |
|  | | | | | | | | | | | |
| Vascular disorders | | 1 (6.7) | | | | 3 | | 1 (11.1) | 1 | 2 (8.3) | 4 |
| Flushing | | 1 (6.7) | | | | 3 | | 0 (0.0) | 0 | 1 (4.2) | 3 |
| Hypertension | | 0 (0.0) | | | | 0 | | 1 (11.1) | 1 | 1 (4.2) | 1 |
|  | | | | | | | | | | | |
| Gastrointestinal disorders | | 0 (0.0) | | | | 0 | | 1 (11.1) | 1 | 1 (4.2) | 1 |
| Increased salivation | | 0 (0.0) | | | | 0 | | 1 (11.1) | 1 | 1 (4.2) | 1 |
|  | | | | | | | | | | | |
| Investigations | | 0 (0.0) | | | | 0 | | 1 (11.1) | 1 | 1 (4.2) | 1 |
| COVID-19 virus test positive | | 0 (0.0) | | | | 0 | | 1 (11.1) | 1 | 1 (4.2) | 1 |
|  | | | | | | | | | | | |
| Metabolism and nutrition disorders | | 1 (6.7) | | | | 1 | | 0 (0.0) | 0 | 1 (4.2) | 1 |
| Hypercholesterolemia | | 1 (6.7) | | | | 1 | | 0 (0.0) | 0 | 1 (4.2) | 1 |
|  | | | | | | | | | | | |
| Renal and urinary disorders | | 0 (0.0) | | | | 0 | | 1 (11.1) | 1 | 1 (4.2) | 1 |
| Kidney stone | | 0 (0.0) | | | | 0 | | 1 (11.1) | 1 | 1 (4.2) | 1 |
|  | | | | | | | | | | | |
| Respiratory, thoracic, and mediastinal disorders | | 1 (6.7) | | | | 1 | | 0 (0.0) | 0 | 1 (4.2) | 1 |
| Dyspnea | | 1 (6.7) | | | | 1 | | 0 (0.0) | 0 | 1 (4.2) | 1 |
|  | | | | | | | | | | | |
| Surgical and medical procedures | | 1 (6.7) | | | | 1 | | 0 (0.0) | 0 | 1 (4.2) | 1 |
| Eye laser surgery | | 1 (6.7) | | | | 1 | | 0 (0.0) | 0 | 1 (4.2) | 1 |
|  | | | | | | | | | | | |
| **Serious adverse events** | | | |  |  |  |  |  |  |  |  |
| Respiratory, thoracic, and mediastinal disorders | | | | 1 (6.7) | 1 | | | 0 (0.0) | 0 | 1 (4.2) | 1 |
| Dyspnea | | | | 1 (6.7) | 1 | | | 0 (0.0) | 0 | 1 (4.2) | 1 |

**Table S4. Laboratory parameters**

|  | **Baseline** | | **EOS** | |
| --- | --- | --- | --- | --- |
| **Characteristic** | **HB-adMSC**  **N = 15** | **Placebo**  **N = 9** | **HB-adMSC**  **N = 15** | **Placebo**  **N = 9** |
| **Comprehensive Metabolic Panel** | | | | |
| Glucose (mg/dL), M (SD) | 99.7 (20.78) | 100.4 (16.96) | 99.9 (22.92) | 100.9 (5.49) |
| Blood Urea Nitrogen (mg/dL), M (SD) | 16.6 (3.61) | 16.0 (4.85) | 16.5 (5.74) | 17.1 (3.69) |
| Creatinine (mg/dL), M (SD) | 0.898 (0.1243) | 0.910 (0.2579) | 0.867 (0.1890) | 0.828 (0.2149) |
| Estimated glomerular filtration rate (mL/min/1.73), M (SD) | 80.7 (11.26) | 81.9 (16.07) | 87.1 (15.14) | 91.8 (14.09) |
| Urea Nitrogen/Creatinine Ratio, M (SD) | 18.5 (4.49) | 18.0 (5.07) | 19.3 (6.83) | 22.4 (10.06) |
| Sodium (mmol/L), M (SD) | 141.5 (1.88) | 142.2 (1.64) | 141.5 (2.07) | 141.2 (1.09) |
| Potassium (mmol/L), M (SD) | 4.29 (0.287) | 4.31 (0.190) | 4.31 (0.285) | 4.30 (0.180) |
| Chloride (mmol/L), M (SD) | 104.5 (1.41) | 104.4 (2.19) | 104.2 (2.24) | 104.3 (1.32) |
| Carbon Dioxide, Total (mmol/L), M (SD) | 25.7 (2.28) | 26.2 (2.54) | 25.7 (1.28) | 26.0 (2.24) |
| Calcium (mg/dL), M (SD) | 9.63 (0.410) | 9.56 (0.357) | 9.72 (0.363) | 9.49 (0.448) |
| Protein, Total (g/dL), M (SD) | 7.11 (0.391) | 6.96 (0.520) | 7.09 (0.329) | 6.82 (0.390) |
| Albumin (g/dL), M (SD) | 4.51 (0.225) | 4.53 (0.278) | 4.49 (0.289) | 4.49 (0.190) |
| Globulin (g/dL), M (SD) | 2.61 (0.328) | 2.42 (0.497) | 2.59 (0.320) | 2.33 (0.412) |
| Albumin/Globulin, Ratio (g/dL), M (SD) | 1.75 (0.236) | 1.96 (0.467) | 1.77 (0.292) | 1.99 (0.431) |
| Bilirubin, Total (mg/dL), M (SD) | 0.46 (0.150) | 0.46 (0.124) | 0.51 (0.177) | 0.54 (0.283) |
| Alkaline Phosphatase (IU/L), M (SD) | 63.9 (17.33) | 75.7 (17.79) | 66.2 (21.61) | 75.6 (15.48) |
| Aspartate aminotransferase (SGOT) (IU/L), M (SD) | 20.3 (6.80) | 20.3 (6.32) | 23.6 (9.03) | 21.1 (5.33) |
| Alanine aminotransferase (SGPT) (IU/L), M (SD) | 14.13 (8.007) | 18.31 (11.221) | 15.26 (9.231) | 18.99 (13.223) |
| **Hematology and Coagulation** | | | | |
| Basophils (%) | 0.79 (0.910) | 0.71 (0.732) | 1.08 (0.820) | 0.87 (0.975) |
| Eosinophils (%) | 1.52 (1.955) | 1.68 (1.627) | 1.49 (1.237) | 1.50 (1.502) |
| Hematocrit (%) | 40.01 (4.052) | 41.63 (3.403) | 41.13 (5.390) | 42.08 (4.157) |
| Hemoglobin (g/dL) | 13.57 (1.435) | 14.16 (1.041) | 13.89 (1.756) | 14.10 (1.474) |
| Lymphocytes (%) | 32.35 (7.020) | 33.33 (9.462) | 32.29 (8.382) | 32.17 (7.656) |
| Erythrocyte Mean Corpuscular Hemoglobin (pg) | 30.80 (1.838) | 30.08 (1.949) | 30.74 (1.572) | 29.76 (1.636) |
| Erythrocyte Mean Corpuscular Volume (fL) | 90.83 (4.404) | 88.30 (3.508) | 90.95 (4.778) | 88.83 (4.101) |
| Monocytes (%) | 7.33 (2.609) | 6.73 (4.615) | 6.84 (2.396) | 8.54 (2.633) |
| Neutrophils (%) | 58.01 (7.031) | 57.54 (9.932) | 58.30 (9.030) | 56.92 (7.372) |
| Platelets (10^9^ cells/L) | 243.3 (52.38) | 209.4 (44.46) | 251.5 (49.19) | 210.2 (48.05) |
| Erythrocytes (10^12^ cells/L) | 4.425 (0.5909) | 4.723 (0.4661) | 4.534 (0.6407) | 4.748 (0.5602) |
| Erythrocytes Distribution Width (%) | 12.39 (0.491) | 12.64 (0.590) | 12.55 (0.538) | 12.87 (0.474) |
| Leukocytes (10^9^ cells/L) | 5.83 (1.891) | 5.47 (1.267) | 5.92 (1.508) | 5.36 (1.051) |
| Prothrombin International Normalized Ratio (Ratio) | 0.97 (0.049) | 0.96 (0.073) | 0.98 (0.041) | 0.96 (0.053) |
| Partial Thromboplastin Time (sec) | 29.85 (2.263) | 31.00 (9.844) | 29.59 (2.895) | 29.63 (2.691) |
